# Ecological strategies shape the insurance potential of biodiversity

**DOI:** 10.3389/fmicb.2012.00432

**Published:** 2013-01-04

**Authors:** Miguel G. Matias, Marine Combe, Claire Barbera, Nicolas Mouquet

**Affiliations:** Institut des Sciences de l'Evolution, UMR CNRS-UM2 5554, Université Montpellier 2Montpellier cedex 05, France

**Keywords:** insurance, biodiversity, functioning, evolution, bacterial microcosms

## Abstract

Biodiversity is thought to provide insurance for ecosystem functioning under heterogeneous environments; however, such insurance potential is under serious threat following unprecedented loss of biodiversity. One of the key mechanism underlying ecological insurance is that niche differentiation allows asynchronous responses to fluctuating environments, although the role of different ecological strategies (e.g., specialists vs. generalists) has yet to be formally evaluated. We present here a simple experimental study that illustrates how different ecological strategies (i.e., generalists vs. specialists) can shape the biodiversity-insurance relationship. We assembled microcosm of generalists and specialist bacteria over a gradient of salinity and found that, bacterial communities made up of generalists were more productive and more stable over time under environmental fluctuations. We discuss our results in context with simple theoretical predictions and propose future directions for biological insurance theory. We argue that beyond species richness itself, it is essential to incorporate the distribution of ecological strategies across relevant environmental gradients as predictors of the insurance potential of biodiversity in natural ecosystems.

## Introduction

Despite ecologists long interest in the role of environmental heterogeneity on the evolution and stability of natural communities (e.g., Hutchinson, 1959; MacArthur and Levins, 1967; Levins, 1968), unprecedented global changes in biodiversity (e.g., McKinney, 1998; Purvis et al., 2000; Duffy, 2003) have prompted a renewed focus in understanding the mechanisms underlying species' responses to increasingly unpredictable natural environments (reviewed by McCann, 2000; Cottingham et al., 2001). The last decade has seen a range of theoretical and empirical studies proposing statistical (e.g., Doak et al., 1998) and biological (e.g., Tilman, 1999; Yachi and Loreau, 1999) mechanisms to explain how biodiversity might determines the stability of natural communities (McCann, 2000; Cottingham et al., 2001). In general, it is well established that more diverse communities should cope better with environmental heterogeneity given that different species will have different responses thus stabilizing the aggregate community properties (Cottingham et al., 2001), although recent synthesis have established that these stabilizing effects may depend on trophic complexity (Jiang and Pu, 2009).

One of these stabilizing mechanisms is the *insurance hypothesis*—species that might be functionally redundant in the ecosystem, increase in numbers in more favorable conditions to compensate for the reduction in performance of the dominant species, thus providing “insurance” for community productivity (Yachi and Loreau, 1999). Biodiversity promotes greater insurance when communities are made up of species that are better performers in different environments (i.e., specialists), so that their responses to environmental fluctuations are asynchronous, hence stabilizing the ecosystem and maximizing productivity (Yachi and Loreau, 1999; Loreau et al., 2003). In variable environments, communities with a greater numbers of species are expected to (1) be more productive because different species are responsible for community productivity under different environmental conditions and (2) be more stable since species compensate each other stabilizing community productivity in time. In practical terms assemblages with higher numbers of species will have higher temporal mean productivity and lower temporal variation in measures such as productivity (measured as the CV; Yachi and Loreau, 1999; Loreau et al., 2003).

The theoretical basis of ecological insurance theory is relatively well established in the literature (Yachi and Loreau, 1999; Norberg et al., 2001; Loreau et al., 2003; Gonzalez et al., 2009), although there is need for further empirical evidence that formally tests its predictions and basic assumptions (Boles et al., 2004; Cooper et al., 2005; Leary and Petchey, 2009; Bouvier et al., 2012). One of the key underlying assumption is that niche differentiation will maximize species' asynchronous responses of species to environmental fluctuations thus insuring community productivity (Yachi and Loreau, 1999). Natural communities are, however, made of species with different degrees of ecological specialization (i.e., specialist and generalist species; Futuyma and Moreno, 1988; Devictor et al., 2010). Until recently, the role of different ecological strategies had been ignored in experiments exploring the relationship between species richness and ecosystem functioning (BEF), even though there is widespread evidence that specialist species have greater extinction risks which makes them more vulnerable to global changes (McKinney and Lockwood, 1999; Devictor et al., 2008). Recent empirical and theoretical studies have revealed that communities made of generalists are more productive on average because of their superior ability to exploit the environmental heterogeneity, although the slope of the BEF relationship is, actually, higher when assemblages are made solely of specialists due to enhanced niche complementarity (Gravel et al., 2011). Despite this evidence, it is not known whether communities made of specialists are more likely to provide greater insurance for community aggregate properties (e.g., productivity) than generalist species.

**Figure 1:**
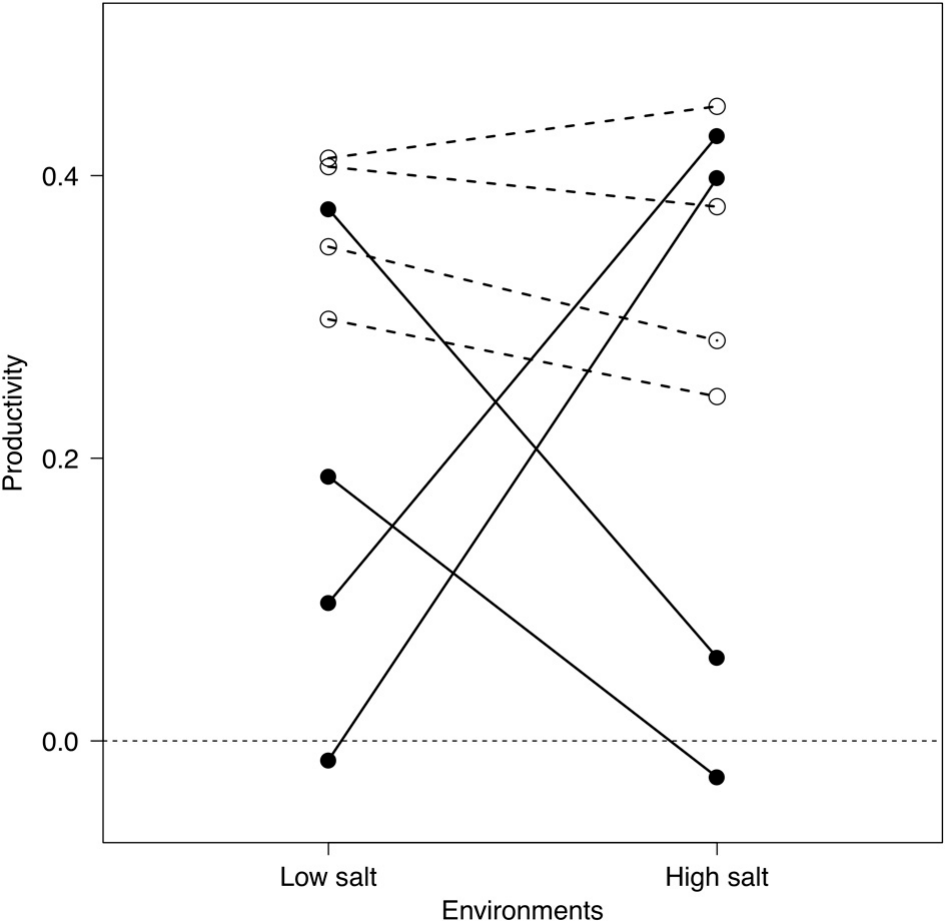
**Reaction norms of generalist (white circles) and specialist (black circles) bacterial strains to different environments.** Reported values indicate the change in optical density after 48 h (as a proxy of productivity) of each strain in monocultures at low- (3 g l^−1^) or high-salt (80 g l^−1^) environments (each symbol indicates the average of 12 microcosms; see “Methods” section).

Depending on the costs associated with being a generalist, it is likely that assemblages of specialists or generalists have different performances across the range of environmental conditions encountered in a fluctuating environment (sensu Kassen, 2002). The magnitude of the insurance effect of biodiversity should therefore be determined by the distribution of strategies within an assemblage of species. We present here a simple experimental study to investigate the relationship between species richness and functioning (e.g., Bell et al., 2005) in communities made up of specialists or generalists with a different numbers of bacterial strains (i.e., 1, 2, or 4). We used bacterial strains, collected across a natural salinity gradient from fresh water to marine environments, to make up bacterial assemblages with different levels of richness for both generalist and specialist strategies over the salinity gradient (Figure 1). The insurance effects were determined by measuring temporal mean community productivity and its temporal variability under fluctuating environments by manipulating salinity in bacterial batch cultures.

## Methods

### Bacterial isolation and niche profiles

We used a pool of bacterial strains that were collected from a range of locations with different salinity conditions. Salinity is a determinant environmental filter influencing the composition of microbial communities (e.g., Lozupone and Knight, 2007), therefore an appropriate trait to evaluate responses to environmental fluctuations. We isolated marine or freshwater bacterial strains from samples of 50 ml of water taken from coastal lagoons or rivers nearby Montpellier (South of France) in several dates in 2009 and 2010. The salinity of these water samples ranged from freshwater (1.2 g/l) to high salinity (>100 g/l). Freshwater samples were spread on previously autoclaved (20 min at 121°C) LB agar plates; samples from coastal lagoons were spread on marine agar plates (BD Difco Marine Agar, 2216); and high-salt lagoons were spread on MB XS (salinity 50 g/l). All of these plates were grown for 5 days at 20°C. Colonies with distinct morphotypes (i.e., size, shape, and color) were isolated, clean-streaked three times, and frozen in glycerol at −80°C.

Salinity profiles were determined for each strain by measuring the bacterial growth across for a range of salinity between 1.2 g/l and 100 g/l. Marine bacteria (optimum growth at salinity >30 g l^−1^) were grown overnight for 24 h at 20°C in 5 ml MB medium under constant orbital shaking (200 r.p.m) in humid chambers. Freshwater bacteria (optimum growth at salinity <5 g l^−1^) were grown in LB standard medium (Luria–Bertani medium; 5 g l^−1^ yeast extract + 10 g l^−1^ tryptone + 5 g l^−1^ NaCl, autoclaved 20 min at 121°C) under the same culture conditions. Cultures were centrifuged (5 min at 3500 r.p.m.) and the supernatant was completely removed. The cell abundances of each strain were then adjusted to match the mean abundances across all strains that were previously measured using light absorbance at 590 nm on a FLUOstar Optima spectrophotometer (BMG LABTECH) in microplates with 200 μ l of overnight cultures of each strain (*n* = 3). This adjustment was done by either diluting or concentrating each overnight using buffered M9 minimal salts (0.1 g l^−1^ NH_4_Cl, 6 g l^−1^ Na_2_HPO_4_, 3 g l^−1^ KH_2_PO_4_, 0.5 g l^−1^ NaCl). This procedure ensured that all bacterial strains had equivalent initial abundances. Finally, bacterial growth was measured in microplates by transferring 20 μl of overnight cultures of each strain in to 200 μl wells already containing 180 μl of each type of medium across the gradient (*n* = 3). Following initial inoculation, we estimated initial abundances using light absorbance at 590 after 48 h.

From a pool of over 250 potential strains, we identified low-salt specialists, high-salt specialists, and generalists (i.e., similar bacterial productivity at high and low salinities; see Figure 1) based on variation between productivity between high- and low-salt medium concentrations. Strains that showed great variation were considered specialists; strains with low variation between the two environments were considered generalists. Finally, we chosen 8 bacterial strains to be used in the experiment (see “Appendix Methods: Model Description” Table A1 for GenBank accession numbers and provisional taxonomical information) based on their consistent reaction norms across several preliminary trials (Figure 1). The specialists' group consisted in bacterial strains that were consistently better performers in either low- or high-salt environments. The pool of generalists consisted of four strains that had similar performances at high- and low-salt concentration.

### Community assemblages

We generated assemblages of bacterial strains with three levels of richness (i.e., 1, 2, and 4 strains) for each of the two experimental groups (i.e., generalists or specialists; Figure 1). Bacterial strains were grown overnight and initial abundances were adjusted as described in the previous section. Bacterial strains were assembled in a “master” 96-well, 1-ml sterile microplate for a total of 11 different community types that consisted of four monocultures, six 2-strain cultures and one mixture with all four strains of either generalists or specialists. The relative proportion of different strains in multi-strain assemblages was kept constant (i.e., 2 and 4 strains).

### Environmental fluctuations

We created a fluctuating environment by manipulating the salinity in bacterial batch cultures; at each transfer, we changed the salinity of the *target medium* (that is, the medium with concentration assigned by the salt treatment for that transfer) by transferring cultures between low-(3 g l^−1^) and high-salt (80 g l^−1^). The basic media was standard LB medium with 5 g of select yeast extract, 10 g of tryptone and 1l sterile water. We used diluted LB medium with in M9 to get LB ½ and added 1.2 g NaCl/l that optimized the growth of these bacterial strains. The salt concentration was manipulated to obtain LB½ [3] (LB ½ + 0.24 g NaCl/ 100 ml solution) and LB½ [80] (LB ½ + 7.88 g NaCl/ 100 ml solution). The experiment was ran for 5 transfers that comprised two full environmental fluctuations.

Twelve replicates of each of the 11 combinations were randomly assigned to six 96-well, 0.25 ml sterile microplates previously filled with the two different medium. To account for the potential variability associated with initial conditions (i.e., high or low salt at *t* = 0), we started six replicates at the low-salt concentration and six at the high-salt concentration. Each well was inoculated with 20 μl of each assemblage already containing 180 μl of the target medium. Following initial inoculation, we estimated initial abundances using light absorbance at 590 nm after 48 h. All microplates were incubated at 20°C in humid chambers for 48 h at which point final abundances were estimated. The difference between the initial and final abundances (i.e., after 48) was used to estimate productivity of each assemblage (Gravel et al., 2011; Jousset et al., 2011; Münkemüller et al., 2012). All assemblages were transferred (20 μl) in to new microplates containing 180 μl of the salt medium concentration corresponding to the subsequent transfer. Preliminary trials confirm that this procedure ensures limited modification of the imposed salt concentrations (i.e., <2%). The whole experiment consisted of 2 (strategies) × 11 (assemblages) × 5 (transfers) × 12 (replicates) = 1320 microcosms.

### Statistical analysis

We tested the effects of diversity and strategies on temporal mean productivity and CV using an unbalanced ANOVA with Diversity and Strategy as fixed factors. All assemblages with the same diversity of strains were pooled together which lead to an unbalance in the number of replicates in each level of diversity due to uneven numbers of possible combinations. ANOVAs were done on log-transformed productivities. In all analyses, we considered a replicate to be the mean productivity (or CV) of each assemblage from all of five time steps pooled together (i.e., *n* = 12). All statistical analyses and data handling were done using R.

## Results

**Figure 2:**
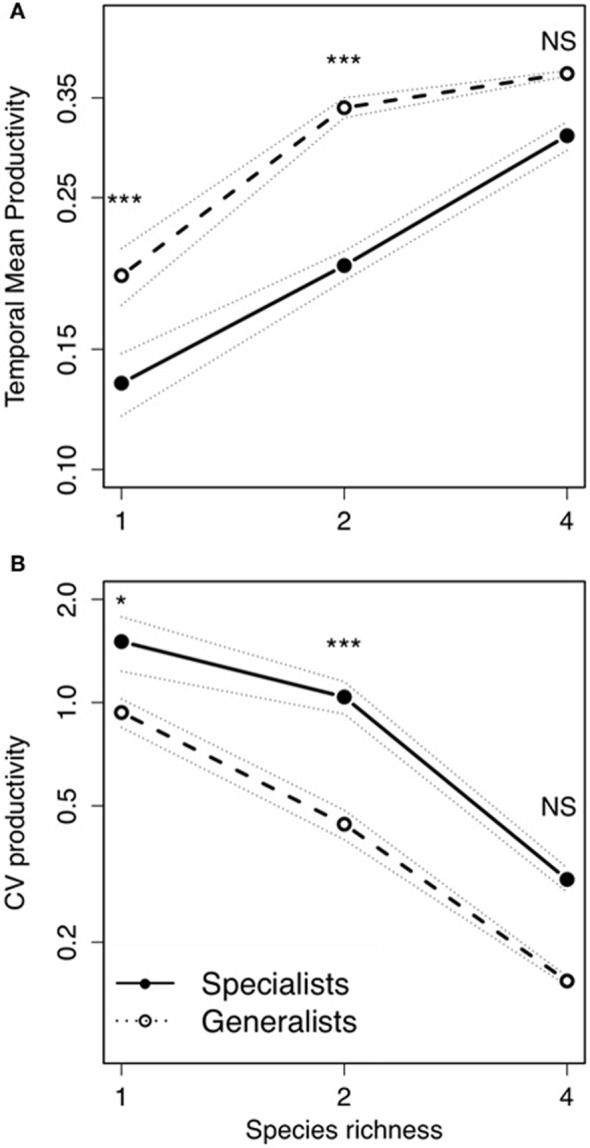
**Bacterial temporal mean productivity and temporal variability in relation to bacterial diversity. (A)** Temporal mean productivity and **(B)** temporal variability (CV) of productivity in fluctuating environments: Each symbol indicates the average of 12 microcosms. Solid lines indicate specialists; and dashed lines indicate generalists. Dotted lines indicate ± standard error of the mean. *Post-hoc* Tukey HSD test to compare means of significant factors with levels of significance: ^*^P < 0.05; ^***^P < 0.001; NS, *P* > 0.05.

Bacterial diversity had a significant positive effect on temporal mean productivity [Figure 2A; *F*_(2, 522)_ = 51.27; *P* < 0.0001; Table 1], although the magnitude of effect was dependent on the strategies making up whether an assemblage was made up of specialists or generalists [Diversity × Strategy interaction: *F*_(2, 522)_ = 5.52; *P* < 0.01]. In contrast, bacterial diversity had a significant and negative effect on the coefficient of variation for mean productivity [CV; Figure 2B; *F*_(2, 522)_ = 12.02; *P* < 0.0001]. Assemblages made up of generalists were generally more productive [Figure 2A; *F*_(1, 522)_ = 77.44.1; *P* < 0.0001], and significantly less variable over time [Figure 2B; *F*_(1, 522)_ = 19.65; *P* < 0.0001]. *Post-hoc* comparisons showed that there were significant differences in temporal mean productivity between specialists and generalists in assemblages with 1 or 2 strains but no differences in mixtures with 4 strains (Tukey HSD test at *P* < 0.05; Figure 2A). The same pattern was found for CV with significant differences in 1 and 2 strain assemblages but not for the more diverse 4 strain assemblages (Figure 2B).

**Table 1:** **ANOVA of the effects of species richness and ecological strategies mean and temporal variability of productivity**.

**Source**	**(a) Temporal mean**				**(b) CV**		
	**Df**	**MS**	***F***	***P***	**MS**	***F***	***P***
Strategy	1	1.43	77.44	*P* < 0.0001	39.37	19.65	*P* < 0.0001
Diversity	2	0.94	51.27	*P* < 0.0001	24.07	12.02	*P* < 0.0001
Diversity × Strategy	2	0.10	5.52	*P* < 0.01	1.04	0.52	*P* > 0.5
Residuals	522	0.02			2.00		

Analysis was done using an unbalanced ANOVA with Strategy and Diversity and main factors. All assemblages with the same species richness were pooled together which lead to an unbalance on the numbers of observations due to the uneven numbers of possible combinations in each level of diversity. The analysis was done using log-transformed productivities. In all analyses, we considered a replicate to be the mean productivity (or CV) across all microcosms of the same assemblage taken from five time steps (i.e., n = 12).

## Discussion

### Insurance effects depends on the ecological strategies

It is crucial to further investigate the mechanisms involved in the emergence and maintenance of the insurance potential of biodiversity to enable better predictions about whether communities will be able to cope with increasingly pervasive landscape homogenization and global climatic change. In particular, since specialist species in are amongst those considered extremely vulnerable under current extinction scenarios (McKinney and Lockwood, 1999; Devictor et al., 2008). Here we have illustrated how insurance effects of biodiversity can indeed be determined by the ecological strategies within a community. Our microcosm experiment showed that bacterial assemblages made up of generalists were significantly less variable over time than those composed of specialists. The effects of bacterial diversity on productivity and temporal variability were consistent across the two types of strategies; although at higher levels of diversity, there was no longer any difference between specialists and generalists. This suggested that the full insurance potential of specialists was only achieved when all species are present (no saturation), which is consistent with predictions that communities made up of specialists (i.e., greater niche differentiation) should have a steeper influence on the BEF relationship than generalists as a result of greater complementary (Gravel et al., 2011).

Previous studies have revealed that the insurance potential of biodiversity is somewhat contingent on species identity, with responses differing depending on the species present in each community (Leary and Petchey, 2009). Similarly, it has also been shown that the insurance effects may vary depending on competitive interactions between species making up community, with more competitively asymmetrical communities buffering the insurance potential of certain communities (Gonzalez and Descamps-Julien, 2004). Overall, these examples emphasize the importance of determining relative contributions of different species to the insurance potential of each community. In fact, in natural communities there is likely a continuum of strategies between “strict” specialists and generalist and that the distribution of these strategies within ecological communities will shape the ecosystem level response to a varying environment. It is thus essential to expand our understanding of ecological insurance beyond the effects of species diversity (Yachi and Loreau, 1999; Loreau et al., 2003; Gonzalez et al., 2009), by incorporating the distribution of ecological traits across the relevant environmental gradients to better predict of insurance potential of biodiversity. Note that the distribution of strategies within the community is likely to mediate the strength of competitive interactions thus determining potential facilitatory or competitive relationships between species that might, in turn, either enhance or weigh down the insurance potential of biodiversity.

### Insurance potential: specialists vs. generalists

In our experiment, the assemblages made of generalist species were significantly more productive than those made of specialist species, which would not have been expected if there was a performance “cost” associated with generalization (Kassen, 2002). In fact, we found little or no trade-off between productivity and specialization, as generalists were often the best performer in each environment (Figure 1). This result might be due to the nature of the procedures we used to isolate bacterial strains that somehow select for particular genotypes. They also illustrate the need for a more comprehensive understanding of the insurance effect that would encompass both the different ecological strategies but also the strength of the cost associated to each strategies.

**Figure 3:**
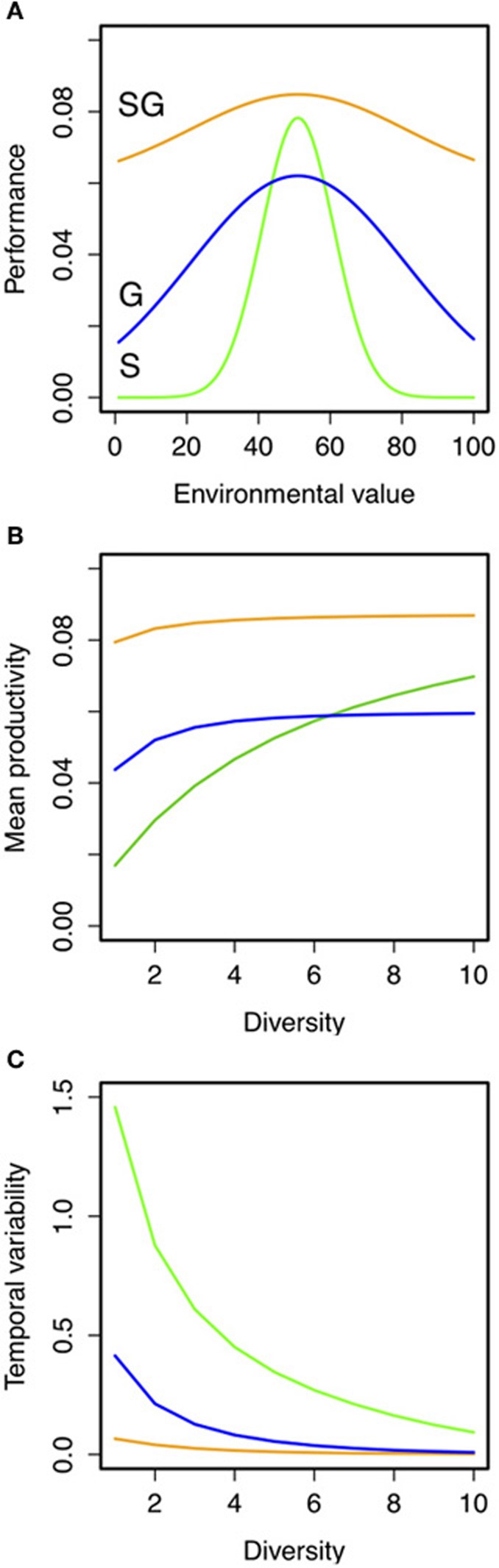
**Hypothetical predictions of the role of ecological strategies (i.e., specialist vs. generalists) on the relationship between species richness, mean, and temporal variability of productivity under fluctuating environments. (A)** Hypothetical niche curves across an environmental gradient for specialist (S, green lines; σ = 10), generalist (G; blue lines; σ = 40) or “super” generalist (SG; orange lines; σ = 40 and maximum productivity of 5; see “Appendix Methods: Model Description” for details). Predictions of **(B)** temporal mean productivity and its **(C)** coefficient of variation were the result of 100 independent simulations for each combination of these parameters. Details on the simulations are given in “Appendix Methods: Model Description” and in Appendix Figures A1–A3.

**Figure A2:**
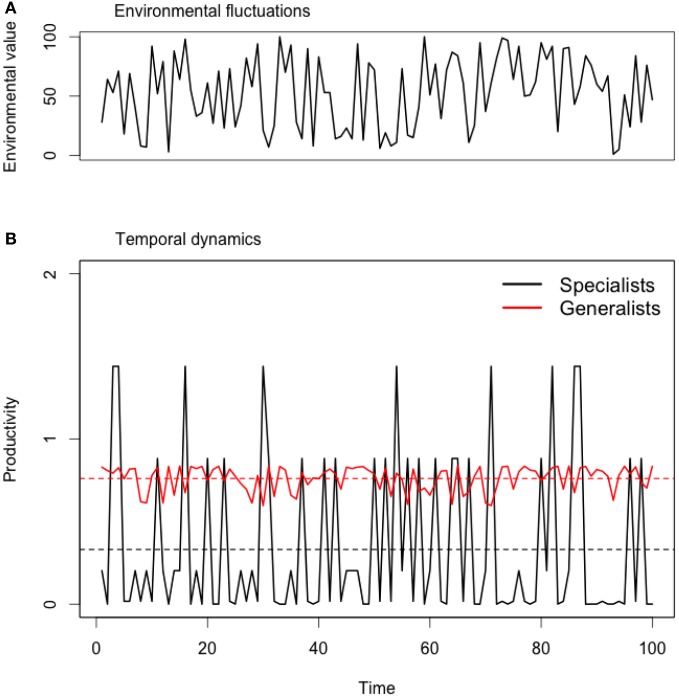
**Temporal dynamics of community productivity of a simulated generalist and specialist species under fluctuating environments. (A)** Sequence of random environmental values ranging between 0 and 100. **(B)** Temporal dynamics of different ecological strategies: specialists (σ = 10) or generalists (σ = 40). For the purpose of illustration, we only present simulations for strong trade-offs (θ = 2). In this particular case, specialists are able to perform better under particular environments than generalists but the overall productivity is lower. This effect is illustrated by the lower mean temporal of specialists (black horizontal lines) when compared to generalists (red horizontal lines).

**Figure A3:**
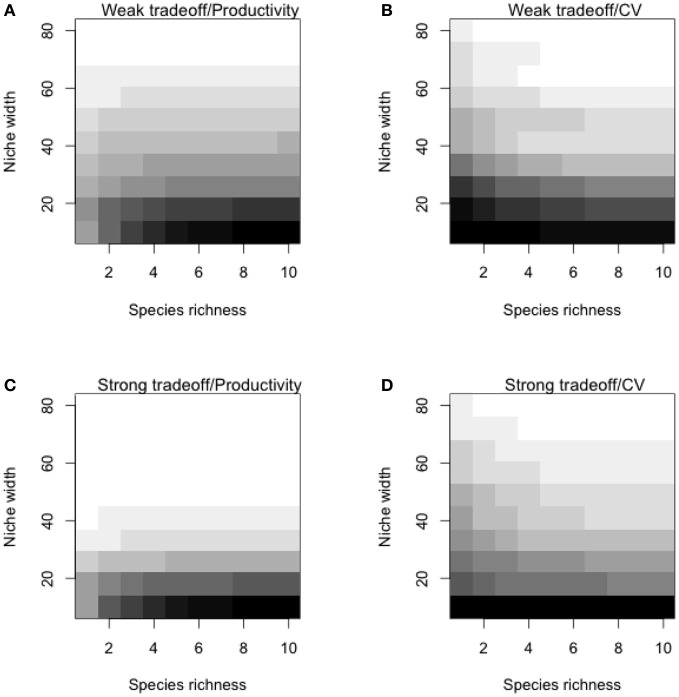
**General theoretical predictions of temporal mean productivity and its coefficient of variation.** Predictions are presented for weak (θ = 0.2; panels **A** and **B**) and strong (θ = 2; panels **C** and **D**) trade-offs. Here, we ran simulations with all combinations of 10 levels of niche width (σ; from 2 to 100) and 10 levels of species richness (from 1 to 10); for each combination of parameters, we ran 100 independent simulations. Additional parameters are explained in detail in the “Appendix Methods: Model Description.” Darker cells indicate greater values of temporal mean productivity or coefficient of variation (CV).

Simple hypothetical predictions can be obtained using a phenomological model of the insurance effects. To do so we built a simple model of community dynamics across a continuum of diversity and strategies, varying the strength of the trade-off between specialization (i.e., niche width) and productivity (Figure 3A; see full description of the model and simulations in “Appendix Methods: Model Description”). We generated theoretical predictions of community productivity and insurance potential of biodiversity for assemblages with different levels of species richness and ecological strategies (see Figures 3B,C; see also Appendix Figure A2). We found that the species richness-temporal variability relationship in assemblages of generalists had higher intercept and slope than those made of specialists (Figure 3C; Appendix Figure A3). Assemblages made up of generalist species were better at insuring community productivity than assemblages made up of specialists that need greater numbers to maintain the insurance potential. Furthermore, the strength of the specialization-productivity trade-off (i.e., the cost paid by the species for being either generalist or specialist) alters the BEF (i.e., reduces the intercept) relationship in assemblages of generalists (as in Gravel et al., 2011). However, the strength of the specialization-productivity trade-off did not have a major effect on species diversity-temporal variability relationship of both strategies (Figure A3), which suggests that the presence/absence of different strategies, rather than the specialization trade-offs, might be determinant for estimating the insurance potential of biodiversity.

More complex models should be used to push our result forward, by including more realistic trade-off curves and different kind of interactions between species; our simple phenomenological model clearly emphasizes the potential for specialist and generalist species to contribute differently to the insurance potential of biodiversity in stabilizing the ecosystems under fluctuations environmental conditions. Our empirical results could not capture the whole range of adaptative strategies we have simulated with our phenomenological model. Our microcosm experiment illustrated a “weak trade-off” scenario that still revealed differences between generalists and specialists in ensuring the stability in productivity throughout the experiment. Future experiments might explore different isolation procedures and investigate other related environmental gradients (e.g., resource availability) that might explain the differences in optimal performance between the different strategies across the different environmental conditions.

### Evolutionary perspectives

We have performed our experiment on ecological time scale (by allowing only 5 batch transfers) but it is likely that a promising future direction will be to include evolutionary response of organisms along environmental gradients. Particularly to consider how an “evolutionary insurance” can emerge in natural communities during diversification and niche differentiation (e.g., Boles et al., 2004). This will imply integrating knowledge on the role of temporal environmental heterogeneity on diversification (Levins, 1968). Generalist or specialist species may be selected depending on the nature of the environmental heterogeneity (e.g., Bell, 1997; Venail et al., 2011), although only particular fluctuations scenarios are likely to promote niche differentiation (e.g., temporal grain; Venail et al., 2011). Moreover, the outcome of local adaptation or selection in complex environments often results neither in specialists nor in generalists but instead in mixtures of overlapping “transient” strategies that might be adapted to certain ranges within an environmental gradient (Barrett et al., 2005). The evolutionary insurance potential of diversity is consequently likely to be the result of different combinations of strategies present at each point during the process of diversification and adaptation. Whether it is driven by strong niche differentiation between specialists or by the evolution of generalists will thus depend on the environmental background in which species evolve. Future research on the insurance will thus have to incorporate the evolutionary history of different species trait diversification to fully understand the potential stabilizing effect of biodiversity on ecosystem functioning (e.g., Gravel et al., 2011).

## Author contributions

Miguel G. Matias designed and performed experiments, analyzed data, and wrote the paper; Marine Combe performed experiments and wrote the paper; Claire Barbera performed experiments; Nicolas Mouquet designed experiments, analyzed data, and wrote the paper. Marine Combe, Claire Barbera, and Nicolas Mouquet collected the bacterial strains.

### Conflict of interest statement

The authors declare that the research was conducted in the absence of any commercial or financial relationships that could be construed as a potential conflict of interest.

## Figures and Tables

**Figure A1:**
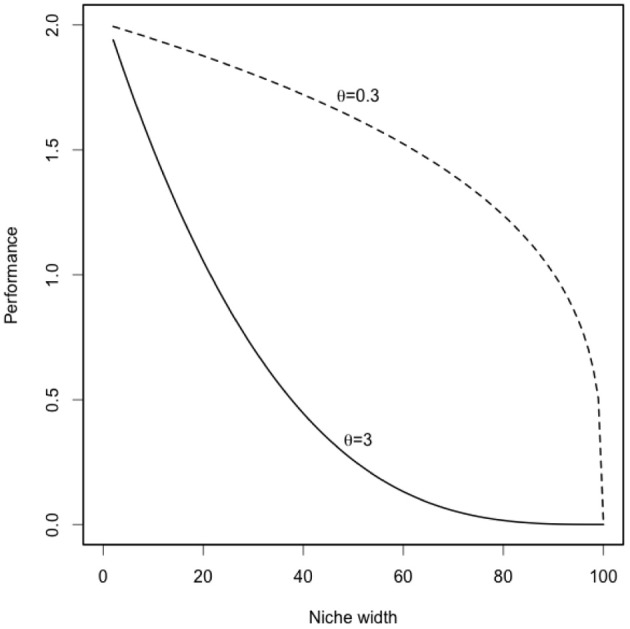
**Trade-off curves between performance and specialization (i.e., niche width).** The parameter niche width determines the ecological strategies of each species (generalists with high σ and specialists with low σ). The parameter θ varies the strength of the trade-off between weak and strong (also called respectively concave and convex) trade-offs (Gravel et al., 2011).

## Appendix

### Appendix methods: model description

We generated theoretical predictions of the role of ecological strategies on the relationship between species richness, mean, and temporal variability of productivity when confronted to a fluctuating environment. These predictions were achieved by (1) generating species niche profiles specialists and generalists with different trade-off strengths and (2) measuring expected community mean productivity and temporal stability of productivity for assemblages of different species richness and combination of strategies.

### Productivity along an environmental gradient

Potential productivity Φ_*ik*_ of species *i* across an environmental gradient *k* was generated following a modified gaussian function:

(1) $$\Phi_{ik}=\Phi_{i}\exp\left(\frac{-{\left(E_{k}/\mu_{i}\right)}^{2}}{{\left(2\sigma_{i}\right)}^{2}\sigma \sqrt{2\times \pi }}\right)$$

where the niche optimum (μ_*i*_) and the environmental value (*E*_*k*_) were set to between 0 and 100 to match the range of salinity used later in the experiment. The parameter σ control for the niche widths (generalists with high σ and specialists with low σ). This function minimizes the differences in overall productivity (sum over the environmental gradient) between the different strategies even though they cannot be made exactly constant (this would be fully true only when this function is integrated between –Inf and +Inf). Examples of the resulting niche curves are shown in main text (Figure 3B).

### Generalist and specialists' performance trade-offs

We implemented the trade-off between species performance and specialization (i.e., niche width) following:

(2) $$\Phi_{i}=\Phi_{m}\times {\left(1-\left(\frac{\sigma_{i}}{100}\right)\right)}^{\theta }$$

where Φ_*i*_ is productivity of species *i*, Φ_*m*_ is maximum productivity; θ is the trade-off strength; σ_*i*_ is the niche width of the species *i* (between 0 and 100). The parameter θ allows us to vary the strength of the trade-off between weak and strong (also called respectively concave and convex) trade-offs (as illustrated in Appendix Figure A1).

### BEF simulations

We assembled all possible combinations from 1 to 10 species and estimated overall productivity for each community using at each time step the productivity of the best performer (Yachi and Loreau, 1999) and averaging these values over time to calculate both the mean temporal productivity and its coefficient of variation (CV). Using the best performer to approximate community productivity makes the assumption that the best performer (i.e., dominant species) is also the best competitor (at that particular environment or resource level; following Tilman, 1999) and that other species do not contribute significantly to the instantaneous community productivity when they are rare (that is when they are not adapted to local environmental conditions). This assumption holds for calculating community productivity in time as in our model species are ought to be productive only when they are adapted to the environmental conditions (which vary in time). Environmental fluctuations were simulated for 10 time steps. At each time step, a random environmental value was chosen between 0 and 100 and we calculated expected productivity of each assemblage (example of dynamics in Appendix Figure A2).

Note that we could have used the sum of the species individual performances within each assemblage but it did not change qualitatively our results (data not shown). Using the mean of species individual performances to calculate a mean temporal productivity would have lead to no relationship between diversity and functioning and thus cannot be used here. Yachi and Loreau (1999) explored the determination of total ecosystem productivity by implementing scenarios of dominance (i.e., total productivity equals the productivity of the best performer) or equivalence (i.e., total productivity is the mean of productivities of all species in mixtures). Equivalence assumes that inter-specific competition is negligible, whilst dominance assumes that the best performer is also the best competitor (at that particular environment or resource level; following the work of Tilman, 1999), which was shown to be a key argument of the insurance hypothesis. These two scenarios are the end points of a continuum and Yachi and Loreau (1999; Figure 2, pp. 1465) have shown that the insurance was operating only mostly in the dominance scenario.

To illustrate contrasting scenarios of specialization, we generated assemblages made of generalists (high niche width: σ = 40), specialists (low niche width: σ = 10) or “super” generalists (σ = 40 with a maximum productivity of 5). These general scenarios were simulated to illustrate different point in the discussion (main text; Figure 3). A more comprehensive set of simulation was then performed on the entire range of levels of specialization (i.e., with values of σ from 2 to 100) under weak and strong trade offs (θ = 0.2 and θ = 2). Predictions of temporal mean productivity and its CV were averaged over 100 simulations for each combination of these parameters. These general simulations showed how species strategies and the strength of the specialization-productivity trade-off alter the BEF relationship and insurance potential. Specialization leads to a steep BEF relationship with high productivities while generalization leads to weaker relationships as generalists cover the entire environmental ranges (Appendix Figures A3A,B). As generalization comes to a cost, maximal productivities of the generalists' assemblages are lower than those of specialists. Assemblages made up of generalists have a greater insurance potential than assemblages made of specialists (lower CV). Although, the extent of this insurance is not affected by the strength of the trade-off (Figures A3C,D). These results show that the insurance potential is more dependent on the width of the niches (specialist vs. generalist) rather than on the maximal species productivity (linked to the strength of the trade-off).

**Table A1:** **Taxonomical information and GenBank accession numbers for all bacteria used**.

**Strategy**	**Strain**	**Code**	**Familiy**	**Genus**	**Accession number**
Specialists	S1	S304	Pseudomonadaceae	Pseudomonas	JX470193
	S2	S287	Pseudomonadaceae	Pseudomonas	JX470194
	S3	S82	Halomonadaceae	Cobetia	JX470195
	S4	S172	Pseudoalteromonadaceae	Pseudoalteromonas	JX470196
Generalists	G1	S136	Vibrionaceae	Vibrio	JX470197
	G2	S328	Pseudoalteromonadaceae	Pseudoalteromonas	JX470198
	G3	S239	Pseudomonadaceae	Pseudomonas	JX470199
	G4	S241	Comamonadaceae	Acidovorax	JX470200

Note that our model is phenomenological and it does not include elements of species interactions and/or resource dynamics; it only illustrates the expected temporal productivities when the distribution of strategies varies in species assemblages. Future developments are required to build on more complex models of species interactions and resources consumption to disentangling the relative contribution of temporal niche differentiation and species interaction to the potential role of biodiversity as insurance (which was not our aim here).

## References

[B1] Barrett R. D. H. MacLean R. C. Bell G. (2005). Experimental evolution of *Pseudomonas fluorescens* in simple and complex environments. Am. Nat. 166, 470–480 10.1086/444440 16224703

[B2] Bell G. A. C. (1997). Experimental evolution in Chlamydomonas. I. Short-term selection in uniform and diverse environments. Heredity 78, 490–497

[B3] Bell T. Newman J. A. Silverman B. W. Turner S. L. Lilley A. K. (2005). The contribution of species richness and composition to bacterial services. Nature 436, 1157–1160 10.1038/nature03891 16121181

[B4] Boles B. R. Thoendel M. Singh P. K. (2004). Self-generated diversity produces “insurance effects” in biofilm communities. Proc. Natl. Acad. Sci. U.S.A. 101, 16630–16635 10.1073/pnas.0407460101 15546998 PMC528905

[B5] Bouvier T. Venail P. Pommier T. Bouvier C. Barbera C. Mouquet N. (2012). Contrasted effects of diversity and immigration on ecological insurance in marine bacterioplankton communities. PLoS ONE 7:e37620 10.1371/journal.pone.0037620 22701572 PMC3373509

[B6] Cooper T. F. Beaumont H. J. Rainey P. B. (2005). Biofilm diversity as a test of the insurance hypothesis. Microbiology 151, 2815–2816 10.1099/mic.0.28026-0 16151192

[B7] Cottingham K. L. Brown B. L. Lennon J. T. (2001). Biodiversity may regulate the temporal variability of ecological systems. Ecol. Lett. 4, 72–85

[B8] Devictor V. Clavel J. Julliard R. Lavergne S. Mouillot D. Thuiller W. (2010). Defining and measuring ecological specialization. J. Appl. Ecol. 47, 15–25

[B9] Devictor V. Julliard R. Clavel J. Jiguet F. Lee A. Couvet D. (2008). Functional biotic homogenization of bird communities in disturbed landscapes. Glob. Ecol. Biogeogr. 17, 252–261

[B10] Doak D. F. Bigger D. Harding E. K. Marvier M. A. Malley R. E. O. Thomson D. (1998). The statistical inevitability of stability-diversity relationships in community ecology. Am. Nat. 151, 264–276 10.1086/286117 18811357

[B11] Duffy J. E. (2003). Biodiversity loss, trophic skew and ecosystem functioning. Ecol. Lett. 6, 680–687

[B12] Futuyma D. J. Moreno G. (1988). The evolution of ecological specialization. Annu. Rev. Ecol. Syst. 19, 207–233

[B13] Gonzalez A. Descamps-Julien B. (2004). Population and community variability in randomly fluctuating environments. Oikos 106, 105–116

[B14] Gonzalez A. Mouquet N. Loreau M. (2009). Biodiversity as spatial insurance: the effects of habitat fragmentation and dispersal on ecosystem functioning, in Biodiversity, Ecosystem Functioning and Ecosystem Services, eds Naeem S. Bunker D. Hector A. Loreau M. Perrings C. (Oxford: Oxford University Press), 134–146

[B15] Gravel D. Bell T. Barbera C. Bouvier T. Pommier T. Venail P. (2011). Experimental niche evolution alters the strength of the diversity-productivity relationship. Nature 469, 89–92 10.1038/nature09592 21131946

[B16] Hutchinson G. E. (1959). Homage to Santa-Rosalia or why are there so many kinds of animals. Am. Nat. 93, 145–159

[B17] Jiang L. Pu Z. (2009). Different effects of species diversity on temporal stability in single-trophic and multitrophic communities. Am. Nat. 174, 651–659 10.1086/605961 19775227

[B18] Jousset A. Schmid B. Scheu S. Eisenhauer N. (2011). Genotypic richness and dissimilarity opposingly affect ecosystem functioning. Ecol. Lett. 14, 537–545 10.1111/j.1461-0248.2011.01613.x 21435139

[B19] Kassen R. (2002). The experimental evolution of specialists, generalists, and the maintenance of diversity. J. Evol. Biol. 15, 173–190

[B20] Leary D. J. Petchey O. L. (2009). Testing a biological mechanism of the insurance hypothesis in experimental aquatic communities. J. Anim. Ecol. 78, 1143–1151 10.1111/j.1365-2656.2009.01586.x 19594662

[B21] Levins R. (1968). Evolution in Changing Environments: Some Theoretical Explorations. Princeton, NJ: Princeton University Press

[B22] Loreau M. Mouquet N. Gonzalez A. (2003). Biodiversity as spatial insurance in heterogeneous landscapes. Proc. Natl. Acad. Sci. U.S.A. 100, 12765–12770 10.1073/pnas.2235465100 14569008 PMC240692

[B23] Lozupone C. A. Knight R. (2007). Global patterns in bacterial diversity. Proc. Natl. Acad. Sci. U.S.A. 104, 11436–11440 10.1073/pnas.0611525104 17592124 PMC2040916

[B24] MacArthur R. Levins R. (1967). Limiting similarity convergence and divergence of coexisting species. Am. Nat. 101, 377–385

[B25] McCann K. S. (2000). The diversity-stability debate. Nature 405, 228–233 10.1038/35012234 10821283

[B26] McKinney M. L. Lockwood J. L. (1999). Biotic homogenization: a few winners replacing many losers in the next mass extinction. Trends Ecol. Evol. 14, 450–453 10.1016/S0169-5347(99)01679-1 10511724

[B27] McKinney R. L. (1998). On predicting biotic homogenization: species-area patterns in marine biota. Glob. Ecol. Biogeogr. 7, 297–301

[B28] Münkemüller T. de Bello F. Meynard C. N. Gravel D. Lavergne S. Mouillot D. (2012). From diversity indices to community assembly processes: a test with simulated data. Ecography 35, 468–480

[B29] Norberg J. Swaney D. P. Dushoff J. Lin J. Casagrandi R. Levin S. A. (2001). Phenotypic diversity and ecosystem functioning in changing environments: a theoretical framework. Proc. Natl. Acad. Sci. U.S.A. 98, 11376–11381 10.1073/pnas.171315998 11535803 PMC58737

[B30] Purvis A. Agapow P. M. Gittleman J. L. Mace G. M. (2000). Nonrandom extinction and the loss of evolutionary history. Science 288, 328–330 10.1126/science.288.5464.328 10764644

[B31] Tilman D. (1999). The ecological consequences of changes in biodiversity: a search for general principles. Ecology 80, 1455–1474

[B32] Venail P. A. Kaltz O. Olivieri I. Pommier T. Mouquet N. (2011). Diversification in temporally heterogeneous environments: effect of the grain in experimental bacterial populations. J. Evol. Biol. 24, 2485–2495 10.1111/j.1420-9101.2011.02376.x 21899638

[B33] Yachi S. Loreau M. (1999). Biodiversity and ecosystem productivity in a fluctuating environment: the insurance hypothesis. Proc. Natl. Acad. Sci. U.S.A. 96, 1463–1468 10.1073/pnas.96.4.1463 9990046 PMC15485

